# Biomechanical Phenotyping of Chronic Low Back Pain: Protocol for BACPAC

**DOI:** 10.1093/pm/pnac163

**Published:** 2022-10-31

**Authors:** D Adam Quirk, Marit E Johnson, Dennis E Anderson, Matthew Smuck, Ruopeng Sun, Robert Matthew, Jeannie Bailey, William S Marras, Kevin M Bell, Jessa Darwin, Anton E Bowden

**Affiliations:** Harvard School of Engineering and Applied Science, Harvard University, Cambridge, Massachusetts; Department of Orthopaedic Surgery, School of Medicine, University of Pittsburgh, Pittsburgh, Pennsylvania; Center for Orthopaedic Studies, Beth Israel Deaconess Medical Center and Harvard Medical School, Boston, Massachusetts; Department of Orthopaedic Surgery, Stanford University School of Medicine, Stanford, California; Department of Orthopaedic Surgery, Stanford University School of Medicine, Stanford, California; Department of Physical Therapy and Rehabilitation Sciences, University of California, San Francisco, California; Department of Orthopaedic Surgery, University of California, San Francisco, California; Department of Integrated Systems Engineering, The Ohio State University, Columbus, Ohio; Department of Bioengineering, Swanson School of Engineering, University of Pittsburgh, Pittsburgh, Pennsylvania; Department of Physical Medicine and Rehabilitation, School of Medicine, University of Pittsburgh, Pittsburgh, Pennsylvania; Department of Mechanical Engineering, Brigham Young University, Provo, Utah, USA

**Keywords:** Low Back Pain, Low Back Disorders, Biomechanics, Human Movement, Motion Analysis Technology

## Abstract

**Objective:**

Biomechanics represents the common final output through which all biopsychosocial constructs of back pain must pass, making it a rich target for phenotyping. To exploit this feature, several sites within the NIH Back Pain Consortium (BACPAC) have developed biomechanics measurement and phenotyping tools. The overall aims of this article were to: 1) provide a narrative review of biomechanics as a phenotyping tool; 2) describe the diverse array of tools and outcome measures that exist within BACPAC; and 3) highlight how leveraging these technologies with the other data collected within BACPAC could elucidate the relationship between biomechanics and other metrics used to characterize low back pain (LBP).

**Methods:**

The narrative review highlights how biomechanical outcomes can discriminate between those with and without LBP, as well as among levels of severity of LBP. It also addresses how biomechanical outcomes track with functional improvements in LBP. Additionally, we present the clinical use case for biomechanical outcome measures that can be met via emerging technologies.

**Results:**

To answer the need for measuring biomechanical performance, our “Results” section describes the spectrum of technologies that have been developed and are being used within BACPAC.

**Conclusion and Future Directions:**

The outcome measures collected by these technologies will be an integral part of longitudinal and cross-sectional studies conducted in BACPAC. Linking these measures with other biopsychosocial data collected within BACPAC increases our potential to use biomechanics as a tool for understanding the mechanisms of LBP, phenotyping unique LBP subgroups, and matching these individuals with an appropriate treatment paradigm.

## Introduction

### Low Back Pain

Low back pain (LBP) is a common condition experienced by 28–42% of individuals in middle adulthood [1, 2]. A hallmark of LBP is the often subjective nature of pain. For many, symptoms of LBP resolve spontaneously; however, a history of LBP is associated with recurrence [3]. In some, LBP symptoms do not resolve and present a chronic challenge. Pain is influenced by a complex mixture of biopsychosocial factors [4], and there has been increased interest in documenting how pain negatively affects an individual's objective function. Periods of LBP can result in living with the burden of disability [1, 5], hallmarked by activity limitations [6–8], which can be objectively measured. These limitations are partially explained by biopsychosocial changes in individuals with LBP that modify how they interact with the world [4, 9–22].

### The National Institutes of Health Back Pain Consortium Research Program

**Figure 1.**
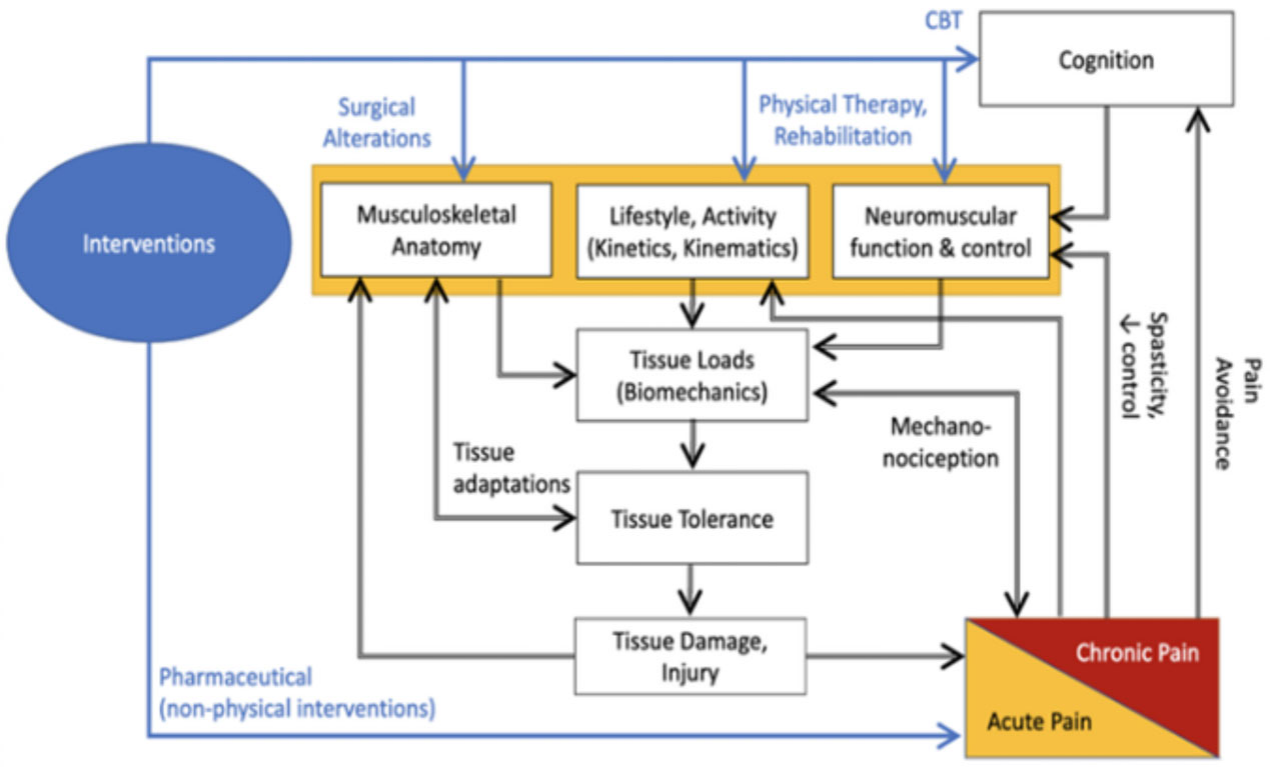
BACPAC Biomechanics Working Group theoretical model of the role of biomechanics in low back pain. This framework posits that the biopsychosocial elements of LBP (in orange) directly and indirectly (via cognition) influence and are influenced by tissue loading during static or dynamic tasks, thereby changing how an individual moves. Knowledge of these interrelationships can assist in proposing targeted interventions (shown in blue).

In 2019, the National Institutes of Health (NIH) Helping to End Addiction Long-term (HEAL) initiative formed the Back Pain Consortium (BACPAC) with the primary objective of informing a precision medicine approach to treating chronic LBP (cLBP) [23]. Described in the anchor article, BACPAC consists of 13 sites across the United States, all working toward the primary objective of investigating an individual’s experience of cLBP through the domains of biology, behavior, and biomechanics [23]. To harmonize data collection and the processing of biomechanical data, the BACPAC Biomechanics Working Group developed a theoretical model (Figure 1), recognizing that all biopsychosocial changes associated with LBP have the potential to modify how an individual tolerates, generates, balances, and responds to tissue loading, which in turn impacts how an individual with LBP executes a movement [24]. Using this framework, we posit that biomechanical measures are representative of a common final framework through which multiple biopsychosocial constructs must interact, potentially providing unique signatures on how individuals adapt to their biopsychosocial strengths and deficits to maintain function.

The Biomechanics Working Group hypothesizes that biomechanical measures provide inherently rich and unique information to help phenotype individuals with LBP. For this reason, many BACPAC research programs include one or more patient-specific biomechanical outcome measures that will be provided to the Data Integration, Algorithm Development and Operations Management Center at the University of North Carolina-Chapel Hill. The purpose of this article is to 1) provide a narrative review of the current understanding of biomechanical outcome measures as potential phenotypic tools and their use in the clinic, 2) describe the technologies used in the consortium to quantify biomechanics, and 3) illuminate the future promise of expanding the phenotypic capabilities of biomechanical outcomes when linking these technologies to direct measurements of other relevant biological, psychological, and sociological factors.

## Methods

Consistent with our theoretical model (Figure 1), we anticipate that all biopsychosocial components of LBP can manifest measurable biomechanical changes (i.e., biomechanical phenotypes). To populate this model, we provide a narrative review of the scientific literature to 1) discuss the definition of a biomechanical outcome measure, 2) provide current evidence that these biomechanical measures can and do describe LBP phenotypes, 3) discuss the factors to consider in the use of biomechanics for phenotyping, and 4) describe the clinical utility of biomechanical outcome measures. After this review, we present the technologies used in BACPAC to address this clinical use case. Finally, we discuss how these consortium-wide measures can be used in an integrated way to improve our understanding of LBP.

## Narrative Review Supporting Biomechanical Phenotypes for LBP

### Definition of Biomechanical Outcome Measures

Biomechanical outcome measures can be divided into three broad categories: analysis of function, performance assessments, and detailed biomechanical analysis (Tables 1 and 2). Ultimately, these outcome measures are interrelated, describing the gross function of movement up to the tissue loads underlying them.

**Table 1.** Broad categories and description of common biomechanical outcome measures

Outcome Measure Type	Description
Analysis of function	Measures of time to complete, maximum capacity, time spent, or other outcome measures that might not fully measure kinematic performance.
Performance assessments	Characterization of the movement patterns “kinematics” of an individual performing a functional task at a single joint or multiple joints.
Detailed biomechanical analysis	Measures of force generators (muscle or electrophysiology) or the effect of forces applied to the body (inverse kinetics, tissue stress and strain).

**Table 2.** Primary outcome measures

Site	Tool	Primary Outcome Measures
Stanford University	Wrist and waist actigraphy	Functional: activity level
		Performance: whole-body activity
Ohio State University	Pelvis- and torso-mounted inertial measurement unit	Functional: lumbar range of motion, rate, symmetry
		Performance: 3D motion of the lumbar spine
Brigham Young University	Skin-adhered wearable stretch sensor array	Functional: lumbar segment range of motion, rate, symmetry
		Performance: 3D motion of individual lumbar segmental angles
University of Pittsburgh	Three torso-mounted inertial measurement units and one thigh-mounted inertial measurement unit	Functional: lumbar range of motion, rate, symmetry, thigh compensation
		Performance: 3D motion of lumbar and thoracic spine
University of California, San Francisco	Kinect and force mat	Functional: body segment range of motion, rate, symmetry
		Performance: 3D motion of body segments, including the torso

#### Analysis of Function

Functional outcome measures capture global or composite metrics of an individual's overall function. Task protocols that analyze function can challenge an individual's strength, balance, coordination, flexibility, aerobic capacity, or some combination of these abilities. Functional task scores often involve single outcome measures (i.e., time to complete, time spent, hold time, maximum motion, or maximal strength) that define cut points for categories of function [25–30]. However, various compensatory strategies might be used to achieve high levels of function, which suggests that two individuals could obtain similar functional scores despite fundamentally different task performances. Composite LBP impairments can manifest as performance deficiency despite a high functional outcome score having been achieved [31].

#### Performance Assessments

Performance outcome measures quantify more deeply how a functional task was executed. Performance assessments can include measuring the kinematics of the spine and other whole-body motions. Many clinicians acknowledge the value of performance assessments yet often rely on skilled observation to document them [32]. Historically, some combinations of standardized protocols and measurement tools have been synergized to measure static postures (goniometers, flex tape, rulers) or maximum movement (inclinometers and dynamometers) [33]. However, tools exist to measure performance with higher precision and resolution than can be obtained with well-trained observation. Researchers and clinicians are gaining access to many tools that can describe dynamic performance in the clinic, accompanied by new promises and challenges on how to use these data.

#### Detailed Biomechanical Analysis

Complementary to measuring dynamic performance, methods exist to explore or estimate how the body produces or balances forces and moments at a joint to contribute to these movements (kinetics). These detailed biomechanical outcomes use kinetic measures, which can be obtained directly from force plates, load cells, or dynamometers or can be predicted from body segment parameters in synergy with direct kinetic measures.

Higher-order biomechanical measurements describe or predict how tissues or tissue systems respond to or generate forces at a joint level. Neurophysiological, radiological, musculotendinous, or neuromuscular measurements can be used as distinct descriptors or in tandem with measures of kinematics and kinetic tools to refine calculations of joint kinetics [34, 35]. These measurements might even aid in understanding the biomechanical impact of movement at a tissue level [36] to explain mechanonociceptive [24] or inflammatory [37] processes relevant to LBP. Despite considerable progress in using detailed biomechanical analysis for phenotyping [38–47], barriers remain to the clinical translation. Thus, subsequent sections will focus on functional and performance outcome measures.

### Evidence for Using Biomechanical Phenotyping for LBP

Scientific literature covers multiple cross-sectional, longitudinal, and prospective studies on biomechanics in individuals with LBP. This section will evaluate the differences in biomechanical function and performance 1) between individuals with and without LBP, 2) in those with more severe patient-reported outcomes, 3) between different established mechanical and psychological biomechanical phenotypes, and 4) as individuals recover from LBP naturally or with therapy.

#### Differences Between Individuals With and Without LBP

Compared with asymptomatic controls, individuals with LBP have different overall function. In large population studies, overall physical activity levels in individuals with LBP follow a U-shaped relationship; too much and too little activity can increase LBP odds [25]. Given that LBP is hallmarked by activity limitations and disability, it is not surprising that individuals with LBP have reduced function compared with asymptomatic controls, as evidenced by 1) reduced static reach [26]; 2) increased time to complete tasks, including Sit to Stand (STS) [27, 28], Timed Up and Go [27], and walking a standard distance [29]; 3) having decreased static balance performance [28]; and 4) trunk muscle weakness, evidenced by decreased strength and increased fatigability [11, 27, 30].

Beyond functional differences, individuals with LBP exhibit performance differences in dynamic movement tasks when compared with asymptomatic controls, specifically: reduced trunk range of motion [43, 48–52], reduced velocity and acceleration [43, 49–58], increased hesitation to initiate motion [59], and altered lumbopelvic coordination [43, 49, 52, 60] such that spine motion is more in phase with hip motion in individuals with LBP than in asymptomatic controls [57]. In addition to differences in lumbopelvic motion, those with LBP have altered or compensatory motion at the thigh, shank, or shoulder when completing complex tasks such as the STS [54, 58, 61]. Ultimately, these functional and performance differences can discriminate between populations with and without LBP with 80–100% accuracy [51, 55, 62–64].

#### Relationship to Patient-Reported Outcomes

Literature to date suggests that both functional and performance-based biomechanical outcome measures are related to an individual’s current level of pain or disability, as captured by patient-reported outcomes and psychological state-trait measures. In many cases, measures of perceived pain, disability, fear, or catastrophizing are associated with increased biomechanical differences within those with LBP, which suggests that differences between those with and without LBP fall along a continuum. Overall, functional outcomes are reduced in individuals with higher levels of pain and disability [28, 65, 66]. Similarly, higher levels of fear-avoidance, catastrophizing, kinesiophobia, anxiety, and depression are also related to functional decrements, including delayed time to complete tasks [65–67].

Performance is also different in populations with higher levels of pain, disability, or fear. Similar to differences between those with LBP and controls, reviews [68, 69] and individual studies have identified that individuals with higher pain, disability, fear-avoidance, pain catastrophizing, and anxiety have reductions in trunk range of motion and movement speed [43, 48, 50, 70–75], increased movement variability [57], delays in initiating movement [59], and more in-phase motion [60, 71, 76, 77].

#### Differences Between Predetermined Biomechanical Subgroups

Individuals with higher levels of pain, disability and fear represent a subpopulation of individuals with LBP. Meanwhile, additional work has attempted to identify whether biomechanical outcomes can discriminate between other predefined LBP subgroups. In general, functional outcome measures have shown a mixed ability to discriminate between populations with different mechanical presentations of LBP [27], including individuals with and without lumbar instability [28]. Unlike mechanical subgroups, there has been some success in discriminating between psychological subgroups. A recent study showed that those with maladaptive responsiveness to pain [78] and magnified psychosocial manifestations of LBP [79] take longer to complete tasks than do individuals who have LBP without these maladaptive signs.

Performance outcome measures have demonstrated considerable promise in identifying mechanical and psychological LBP subgroups. Early literature revealed that low back kinematic measures could distinguish various combined Quebec Task Force presentations of LBP with 56–77% accuracy [51, 55, 62], with higher accuracy for comparing structural vs muscular types of LBP [55, 62]. More recent studies have demonstrated that low back biomechanics can discriminate between individuals with high or low scores through the use of the Keele STarT Back Screening Tool with 65–75% accuracy [64, 80]. Of greatest promise are recent studies showing that biomechanical performance can discriminate between individuals classified as having a clinically observed movement disorder consistent with flexor vs extensor pain patterns with an accuracy of 90–98% [81, 82], which suggests that these measures might enhance clinician objectivity to quantify and classify motions.

#### Differences over Time

Biomechanical measures have been shown to change over time. Overall, as individuals with LBP recover, either spontaneously or after therapeutic interventions, they exhibit improvements in biomechanical functions [7, 29, 83–85] that correlate with decreases in pain and disability [85]. Similarly, biomechanical performance improves over time as individuals with LBP recover [69, 86]. These improvements follow a unique progression of increased trunk range of motion [7, 69, 75, 84, 87, 88], followed by increased movement speed [69, 88], which are related to decreases in pain and disability [69, 71, 86, 88].

It is essential to acknowledge that biomechanical measurements are distinct from changes in patient-reported outcomes. A recent case study has identified that although most (54%) individuals experience improvements in pain, disability, and biomechanical performance simultaneously, a good proportion of individuals also experience biomechanical changes after changes in perceived pain and function (31%) [86]. A longitudinal study demonstrated that by 3 months, objectively measured biomechanical changes lag behind perceived changes in pain and disability [88, 89]. This unique biomechanical recovery can be of considerable importance, as prospective studies have demonstrated that individuals with less biomechanical recovery had increased odds of experiencing low back re-injuries after returning to work [90]. This suggests that biomechanical performance can be an objective measure that might capture lingering vulnerabilities that could lead to higher spinal loading [36].

### Factors to Consider for Biomechanical Phenotyping

Consistent with our theoretical model, biomechanical measures have the face and criterion validity to characterize individuals with LBP. However, our group acknowledges controversial findings, often explained by methodological issues that threaten the criterion, content, and construct validity of the biomechanical measurement and subsequent postprocessing.

#### Description of Study Groups That Threaten Criterion Validity

To date, most biomechanical studies have made observations across small sample sizes (n = 10–30 per group) between LBP and control populations [42, 52, 54, 91], which limits study generalizability and the ability to detect differences between heterogenous control and LBP populations. Asymptomatic control groups can include individuals who have reduced trunk velocity who are more likely to develop future LBP [39], a finding that might be explained by the fact that asymptomatic control participants who have had a history of LBP not only are more likely to experience recurrent LBP (recurrence) but are also more likely to present biomechanical features similar to those with LBP [38, 52, 92, 93]. LBP also represents a heterogenous spectrum, and those with lower levels of pain and disability are potentially less likely to differ from asymptomatic controls [48, 52]. More interesting is current evidence to suggest that there are LBP subgroups that can exhibit nearly opposing biomechanical performance, which, if not accounted for, could diminish the ability to discriminate between those with LBP and those without [69, 72, 76, 91]. These confounders can be reduced by tighter exclusion criteria or description of LBP history and type in larger studies.

#### Selection of Experimental Task Threatens Content Validity

Considerable variation exists in experimental protocols or tasks [42, 94]. However, nearly 50% of experiments do not report the goals or design of their biomechanical tasks [42]. To identify differences between LBP groups or subgroups, it is important to understand whether a task can sufficiently challenge the biopsychosocial elements of LBP. In the literature, it appears that tasks that require greater range of motion [49, 52–54] are more likely to discern differences between individuals with and without LBP than are tasks without (e.g., walking). Challenge also comes from movement speed, where static tasks [72] appear to be less discriminatory than are dynamic tasks, which encourage movement speed [55, 75]. Recently, Wernli et al. demonstrated that a challenging task might be patient specific, showing that perceptual and biomechanical improvements are most likely to occur in the tasks the patient finds most challenging [69, 86]. The most sensitive and specific task protocols remain unknown, but there might be an optimal battery to improve discriminative power [43, 54].

#### Consideration of Construct Validity

Growing technology offers the exciting potential of new measurement tools that can capture dynamic biomechanical performances with low cost, complexity, and training to promote clinical usability. However, the construct validity of measurement tools and postprocessing varies in precision, reliability, and complexity, offering avenues for refinement. Emerging technologies must have sufficient criterion validity to reliably capture their measurements of interest [26, 95, 96]. However, fewer than 25% of experiments demonstrate reliability, and fewer still consider test-retest reliability [42].

To date, with regard to developing a tool for LBP phenotyping, the power of many biomechanical performance methods has been achieved with limited spatial complexity by measuring torso segment angles and lumbopelvic motion [54]. However, tools exist to resolve motion at a vertebral level and/or quantify the performance of other joints that could compensate to maintain function, which could provide promising information. The temporal complexity of tools is equally important, as outcome measures with higher temporal complexity, e.g., velocity and acceleration, have better discriminative power than do discrete values of angular displacement [43, 54, 55, 62]. This capacity to capture higher temporal complexity is possible only because of postprocessing techniques, which can also change the discriminative power of performance-based biomechanics [64]. Knowledge is limited with regard to the spatial, temporal, and processing complexity needed to enhance the discriminative power of performance-based biomechanical outcome measures [42, 54, 69]. Nevertheless, with growing complexity in tools, as well as artificial intelligence and machine learning (ML)–guided signal processing, we anticipate considerable growth toward developing accurate measures that are interpretable for clinicians (e.g., uses of ML are described in the “University of Pittsburgh: Wearable Sensors” and “Brigham Young University: Multisegmented Vertebral Body Level Motion” sections later in this article).

### Clinical Translation

Clinicians express a need to measure biomechanics, identify unique phenotypes, and improve therapy [46, 97, 98]. To date, “quantifying” function has been defined by the functional outcome measure, whereas “quantifying” the performance has been left to the clinician's skilled observations of the patient's performance within the functional outcome measure [97, 99, 100]. Clinicians use functional outcomes to guide clinical decision-making and to provide indicators of improvement or plateau to Centers for Medicare and Medicaid and third-party payers for reimbursement of services. To improve the reliability and validity of skilled observations, clinicians typically rely on clinical classification systems in attempts to subgroup homogenous characteristics of patients with cLBP [94, 99, 101, 102]. However, these systems require immersive training and numerous clinical exposures [103] to recognize various functional limitation situations [97], and clinical inexperience can lead to misdiagnosis and impaired LBP recovery [104, 105].

In BACPAC baseline data collection, clinical and biomechanical harmonization allows clinical functional outcomes to be measured in parallel with biomechanical outcomes to potentially provide future clinical analyses with real-time objective data. Objective biomechanical performance measures could act in tandem with clinicians' observations of biomechanical performance for phenotyping [26]. This synergistic approach might reduce inter-clinician variability, implicit biases, and burdens of reevaluations and variations in patients' care plans. It could also improve abilities to track kinematic records of patients with LBP throughout their therapeutic progressions.

## Results

### Technology Used Within BACPAC

**Figure 2.**
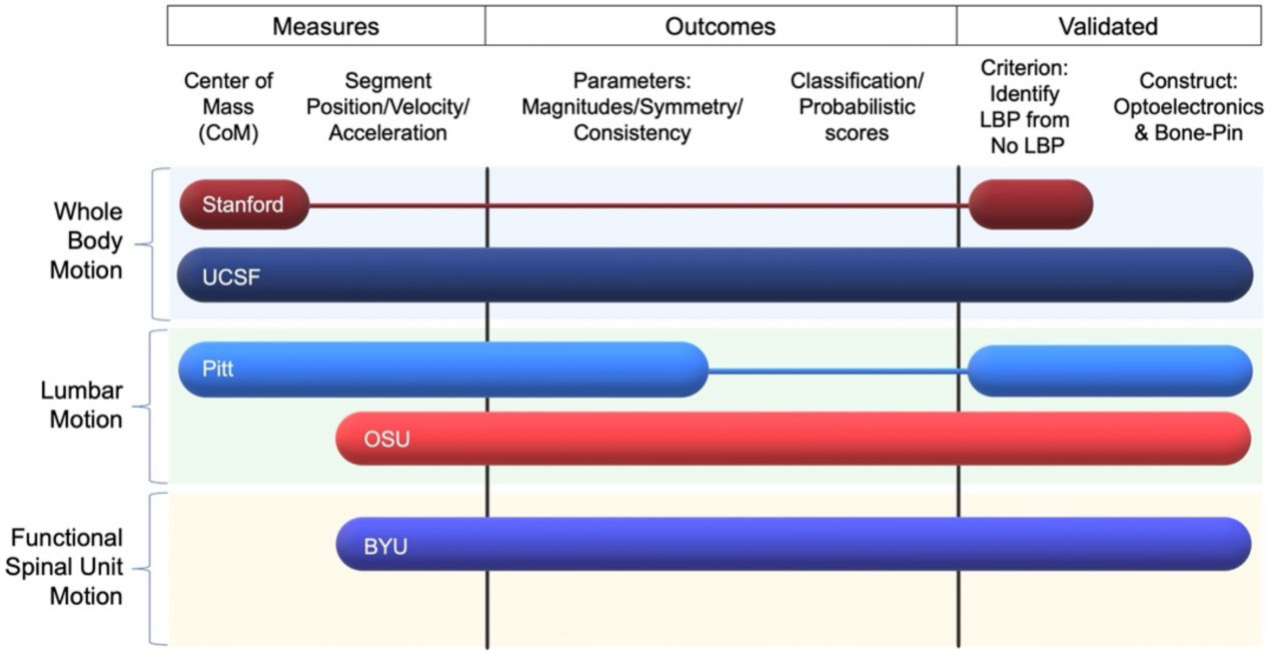
Basic overview and comparison of technologies used by BACPAC sites. In this figure, rows indicate the tool used by each site and the tool's general spatial complexity. Three factors were considered for columns. Measures determined whether the tool acts regionally or describes whole-body motion. Outcomes provide a general overview of the anticipated process and deliver outcome measures to a patient or clinician. Validation provides whether the tool has successfully demonstrated suitable criterion and construct validity.

With the growth of reliable, low-cost, valid, and easy-to-use tools that can be rapidly processed, many of the research sites within BACPAC aim to achieve the reality of integrating objective biomechanics into clinical practice to exploit its potential as a diagnostic, prognostic, and phenotyping tool to aid in precision medicine. Despite each site taking a unique approach, (Figure 2), the overarching aim that guides all sites is to improve the capacity of biomechanics to become a phenotypic tool, especially in analyzing cLBP. This section intends to provide a high-level description of each technology used within BACPAC, along with the goal of the site.

#### University of California, San Francisco: Markerless Motion Capture

**Figure 3.**
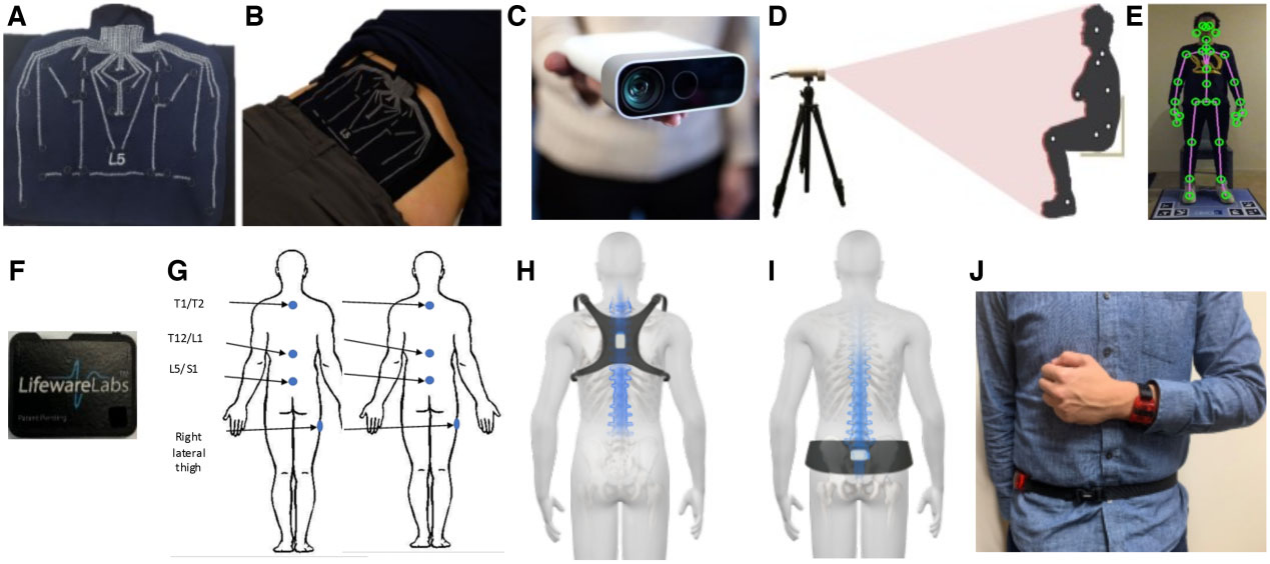
Visual of the technology to measure biomechanical performance included in BACPAC. Plot **(A)** depicts the SPINE Sense System developed by Brigham Young University that **(B)** is adhered onto a participant's skin to track localized lumbar skin strain fields, which correlate with underlying motion of lumbar functional spinal units. The University of California, San Francisco, has leveraged markerless-based kinect cameras **(C)** to estimate joint centers, thus capturing whole-body sagittal plane motion **(D)**, which can be combined with pressure mats **(E)** to calculate whole-body kinetics. The University of Pittsburgh adheres off-the-shelf Lifeware IMUs **(F)** to the skin over the right thigh and various spinous processes **(G)** to measure both hip and lumbopelvic kinematics. Ohio State University has miniaturized their existing lumbar motion monitor mounting accelerometers to a chest-mounted harness **(H)** and pelvis belt **(I)** to quantify lumbopelvic motion with a system that can be worn over the clothes. Using accelerometers to characterize general activity levels, Stanford University uses a dual-mounted accelerometer system **(J)** to determine the best accelerometer placement and activity thresholding to improve the phenotyping capabilities of existing actigraphy.

University of California, San Francisco, the site of the REACH Interdisciplinary Mechanistic Research Center, has created a markerless motion capture-based biomechanical assessment of the STS functional test for LBP. Full-body 3D skeletal tracking motion data are collected with an Azure Kinect depth camera (Microsoft Corp., Redmond, WA, USA) and a custom floor mat for enhanced ankle placement estimation (Figure 3D, 3E). Subjects are coached through a five-times STS task from a user interface application on a laptop. Although many studies have confirmed that LBP significantly increases the STS time, few have explored the compensatory biomechanics adopted by patients with LBP during the STS test. Capturing the 3D motion patterns of multiple joints throughout the body and applying limb length algorithms to generate accurate joint kinematics enables us to apply skeletal and biomechanical models and estimate 1) individual joint and overall postural trajectories, 2) velocities and accelerations of joints and body segments, and 3) joint forces and total mechanical energy estimates. These data types provide a more inclusive view into compensatory movement patterns, beyond spinal kinematics alone. Using a platform that has been validated to gold standard motion capture in a control cohort [95, 106], with high concordance and correlation for joint positions and angles (Lin’s concordance correlation coefficient [CCC]: 0.82–0.99, interclass correlation coefficient [ICC(3,1)]: 0.82–0.99), with low mean absolute error (3–6 degrees, 6.5–13.7 degrees/second). Concordance and correlation in measuring body forces and torques were high (CCC: 0.76–0.95, ICC(3,1): 0.76–0.95).

**Figure 4.**
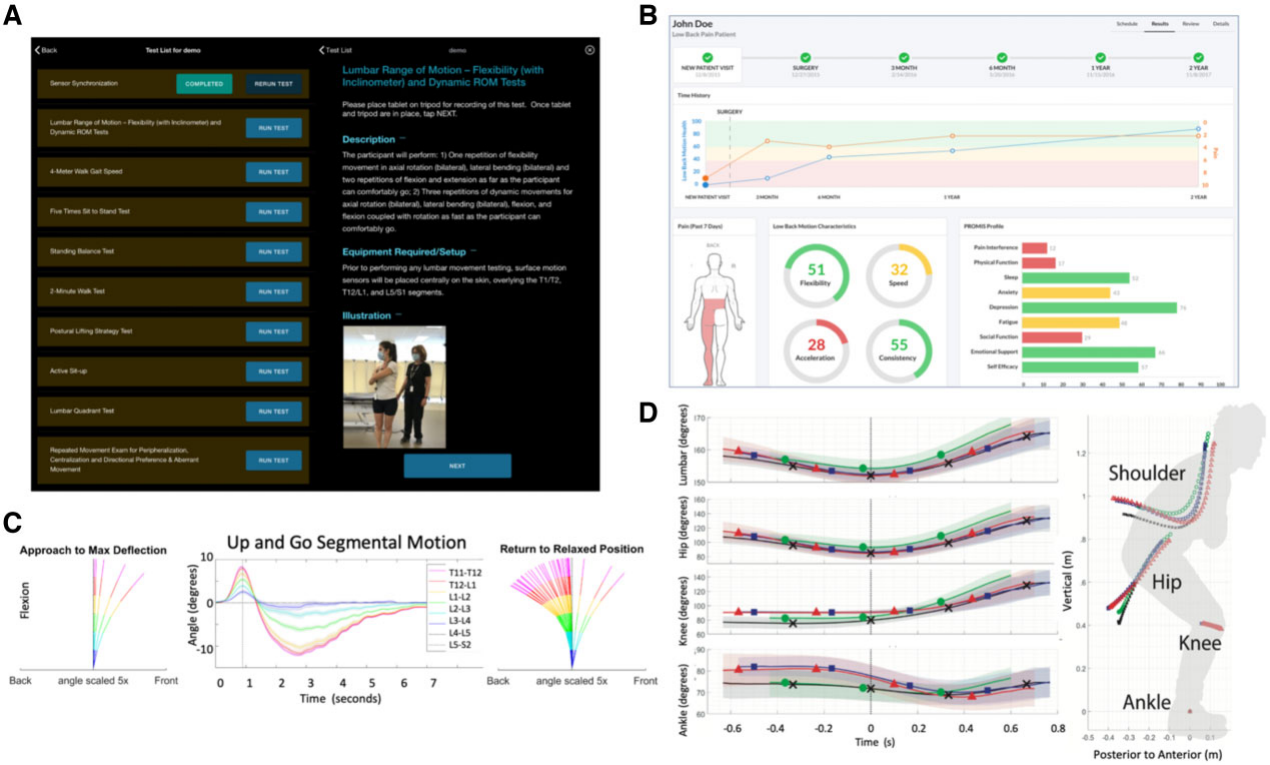
Example of system interfaces. Many system interfaces, such as the LB^3^P developed by University of Pittsburgh, provide an interface to clinicians to select specific tasks (top left). The interface provides a brief task description that can be expanded to include specific instructions to coach participants and therapists through a series of specific exercises **(A)**. Furthermore, many of these systems immediately display measures to the clinician and participant in real time. The interface from Ohio State University **(B)** displays discrete measures of low back motion characteristics of a patient and how they change over time. Brigham Young University captures motion at each lumbar spine segment **(C)**, with the capability to break a complex task such as a timed Up and Go into phases to display how vertebral bodies move when a patient returns to a relaxed position. Systems such as that used by the University of California, San Francisco, provide a technology to allow clinicians to evaluate comprehensive temporal complexity at multiple joints **(D)**, which can be compared between different populations.

As with data provided by marker-based motion capture, this approach offers time-series outcomes for kinematics, kinetics, and dynamics of each joint tracked (Figure 4D). From that, we can use conventional analysis approaches by extracting peak measures of individual variables. However, we can also use the complete time-series data outputs for advanced analytics to capture the complete movement profile and movement quality. We can compare potential differences in compensatory strategies between spine patient groups by using methods like nonlinear principal-components analysis to identify clusters of specific biomechanical variables that correspond with different patient groups. Additionally, we can reduce multi-joint time-series data into full-body postural trajectories comparing features of these trajectories among spine patients.

#### Stanford University: Actigraphy

In partnership with the Mechanistic Research Center at University of California, San Francisco, Stanford University is leveraging the Actigraph (Pensacola, FL, USA) triaxial accelerometer (and other activity trackers) that can capture 3D acceleration (and rotation and orientation in some sensors). Such advancements in sensor integration, combined with advanced data processing algorithms, now allow more insight into the real-life monitoring of whole-body movement, in addition to the standard daily step count estimation. Through their evolution in physical activity monitoring, criteria for standardized methods of accelerometry data collection and processing were established to extract the “gravity unit” and “activity count” that represent accelerations due to body movement [107]. Physical activity metrics (duration, intensity, frequency, pattern) derived from activity count have been widely used over the past two decades, whereas metrics derived from raw acceleration data by open-access algorithms [108] have recently gained popularity for their cross-device comparability. Such measures can be used to further quantify the amount of daily physical activity stratified into standard intervals, including sedentary, light physical activity, and moderate-to-vigorous physical activity.

Although objective physical activity monitoring has been used for mortality prediction and phenotyping of various pathologies, its clinical utility for LBP digital phenotyping has not yet been fully established. Furthermore, because individuals with spine disease often spend most of their nonsedentary time doing light-range physical activity, with little to no moderate-to-vigorous physical activity, traditional accelerometry activity cut points derived from healthy populations to estimate energy expenditure might not be sensitive enough to evaluate the physical activity patterns in LBP populations. To address this issue, a set of activity cut points has been developed (for hip-worn devices) that are tailored to individuals with musculoskeletal pain (called the Physical Performance Intervals) [109] and produce a more granular assessment of light-intensity activity. Stanford’s recent work investigated the potential for physical activity phenotyping in an LBP cohort and identified that sedentary and light physical activity, instead of moderate-to-vigorous physical activity, were sensitive for distinguishing patients with LBP from healthy controls with an 88% accuracy [110].

Recently, wrist-worn devices have gained popularity in both commercial and research use because of improved wear time compliance [111], whereas accelerometers placed on the hip/waist were originally preferred and extensively used, given this location's proximity to the center of mass and its ability to better estimate physical activity intensity and metabolic energy expenditure [112]. However, it is important to note that data derived from the wrist-worn device cannot be directly compared with legacy findings derived from the hip-worn device, as arm movement often does not involve whole-body movement, which can lead to misclassification of activity. To address this issue, both the wrist-worn and hip-worn devices will be used to derive the physical activity profiles (amount/intensity/distribution) of populations with LBP (Figure 3J) to improve the accuracy of wrist-based physical activity estimates.

#### University of Pittsburgh: Wearable Sensors

The University of Pittsburgh LB^3^P Mechanistic Research Center has developed an inertial measurement unit system comprised of two components: 1) a clinic component to gather hip-spine kinematic data during structured functional testing and 2) an at-home component to capture ecological momentary assessments and lumbar spine kinematics over an unstructured at-home test period. Altogether, the entire system is created to seamlessly fit into common clinical practice with minimal disruption to provide actionable objective measures of lumbopelvic motion to inform therapeutic practice.

Clinicians prepare four validated commercially available wireless Bluetooth LE IMUs (Lifeware Labs, LLC, Pittsburgh, PA, USA) (Figure 3F), positioning them on the spine and right lateral thigh (Figure 3G). Clinicians interact with an Android™ smart device (Samsung, Seoul, South Korea) loaded with custom mobile app software (LB^3^P Clinical Toolbox, HARI Labs, University of Pittsburgh, Pittsburgh, PA, USA) to reliably capture participants' biomechanical performance data across a series of common functional tests. Clinicians select standardized functional tests (e.g., five-times STS), follow protocol instructions, and video tests, while sensors simultaneously capture raw data and transmit to the Android™ smart device (Figure 4A). Digitally packaged sensor data are sent to a secure cloud platform for data processing. As development progresses, processed data will be readily viewable in a secure clinician portal, showing participants' spine and lumbopelvic ranges and velocities of motion, normative comparisons, and unique biomechanical markers captured with kinematics, computer vision, and ML. Specifically, efforts are ongoing to develop deep ML algorithms that can correctly identify and characterize motions of the lumbar spine during both clinical and field assessments via supervised ML approaches using video and motion sensor data. Upon verification, these tools have the potential to streamline the phenotyping process, resulting in more objective and clinically translatable metrics.

After clinic testing, the second system component is deployed. Two water-resistant inertial measurement unit sensors (size: 4 cm× 6 cm× 1 cm) with onboard data logging are placed on T12/L1 and L5/S1, while a secure cross-platform ecological momentary assessment app is loaded to participants' smartphones. At home, sensors continuously log data as participants wear them for up to 7 days and move unstructured throughout their days. Participants interact three times a day via the ecological momentary assessment app for a 10- to 15-second survey to capture subjective sleep times, activity intensity, pain intensity, and interference. Participants may enter diary notes and connect with study representatives via secure in-app messaging during the at-home testing period. Future development aims to align at-home kinematic data using ML with subjective ecological momentary assessment and clinic data to establish education on pain-reproducing habitual patterns and plan-of-care development.

#### Brigham Young University: Multisegmented Vertebral Body Level Motion

The Technology Research Site (Tech Site) at Brigham Young University has developed the Spinal Nanosensor Environment (SPINE Sense System; Figure 3A), which is a passive, wearable array of 16 nanocomposite stretch-sensors that provides detailed, quantitative information on the kinematic motion of each lumbar functional spinal unit and wirelessly transmits that information to a nearby smartphone. The system leverages an ML paradigm to identify motion phenotypes and cLBP subphenotypes associated with structural and neurological deficits of the spine. We anticipate that the diagnostic accuracy of the system will be augmented through inclusion of demographic information, as well as annotations of structural information obtained through magnetic resonance imaging. Such a tool is relevant to the diagnosis, monitoring, and follow-up assessment of cLBP.

The nanocomposite stretch sensors used in the SPINE Sense System are comprised of a silicone matrix with dispersed nickel nanostrands and nickel-coated carbon fibers, and they exhibit an inverse piezoresistive response (i.e., the electrical resistance drops dramatically as the sensor is stretched) [113]. The stretch sensors are attached to a kinesiology tape substrate, which is subsequently adhered to the skin of the lumbar region (Figure 3B). The stretch sensor array is attached via a micro–high-definition multimedia interface (HDMI) connection to a custom printed circuit board / battery system that communicates via Bluetooth with a nearby smartphone. The raw material cost of the entire system (not including the reusable printed circuit board or smartphone) is approximately $8.

The sensor arrangement was obtained through an optimization process [114], and a vertebral bone pin cadaver study was used to validate that the skin strains measured by the system correlated with underlying spinal segment motions, achieving root mean square error rates less than 10% [115]. The system was then used to phenotype the lumbar kinematics of a small trial cohort of subjects (12 healthy controls and 10 subjects with cLBP), with a K-nearest-neighbors ML algorithm, and it achieved a 99% classification accuracy.

The SPINE Sense System is designed to be both clinically accessible and immediately useful. Placement of the device requires identification of a single anatomic location (spinous process of the L5 vertebra) (Figure 3B). After the device has been placed on the lumbar skin, an associated smartphone app is used to trigger data collection and subsequent transfer back to the smartphone during a series of repeated diagnostic functional movements. The diagnostic functional movements consist of both single-plane movements (e.g., flexion-extension) and functional movements (e.g., “Up and Go” from a seated position) (Figure 4B). The total time required to both place the sensor array and collect the biomechanics data from the series of diagnostic functional movements is approximately 15 minutes. Usability of the SPINE Sense System prototype (along with its associated smartphone app) was evaluated by 32 panelists (19 clinicians and 13 patients with cLBP) using a standard, validated system design evaluation tool, the System Usability Scale [116], and it demonstrated an “above-average” usability rating.

#### Ohio State University: Spinal Motion Assessment Monitor

The Ohio State University Spine Research Institute (OSUSRI) Tech Site has worked for decades on the innovative use of an individual's motion characteristics captured by wearable motion sensors to quantify functional spine health. The development, validation, and innovative use of wearable motion technologies at OSUSRI to assess spine function have been extensively described in dozens of scientific peer-reviewed journals [36, 55, 56, 62, 88]. Research and development efforts supported by the NIH have allowed OSUSRI to translate decades of research into an integrated cloud-based Spine Health Platform that leverages motion-based metrics, patient-reported outcomes, and other meta biomarkers to facilitate deep patient phenotyping, predict treatment probabilities, and personalize spine care.

Subsequent development has resulted in the creation of a functional low back health assessment system that enables central dashboard access to patient-specific variables that span the biopsychosocial spectrum, including novel measurements of lumbar spine function through wearable motion sensors. The low back motion assessment hardware system consists of two 9-axis inertial measurement unit sensors mounted on the upper back and pelvic harnesses (Figure 3H and 3I, respectively), which are worn over clothing. This system is designed to specifically measure the motion of the lumbar spine in all three axes, with advanced algorithms correcting sensor drift to provide a resolution within 100 microns. The custom harnesses were optimized for comfort, form-fit, and easy sensor placement.

Through the use of a technology platform built on an enterprise-grade Amazon Web Services server, individuals are coached through eight standardized motion tests to assess the extent of an individual's low back impairment. Six of these motions require participants to 1) move to their maximum positions and 2) move as fast as they comfortably can within each of the three cardinal planes of the body. The final two trials evaluate coupled motions and require participants to flex and extend their trunks as fast as they can comfortably while they are twisted to the right or left as far as they are able. The motion tests typically take 10 minutes or less to perform the tasks.

The software is designed to verbally and visually use intuitive graphics and animated videos to guide users and patients through the data collection process step by step, and it includes automatic updates. The system is assisted by a rigorous set of U.S. Food and Drug Administration design controls to ensure patient safety and data quality. Questionnaires can be completed in person or emailed automatically via built-in scheduling functions. Finally, training modules built directly into the software prevent users from collecting data before they have been appropriately trained.

Collected data is automatically processed, analyzed, and available for reporting immediately after collection. Standardized questionnaires are scored, and motion assessment results are compared with those of normative populations for intuitive interpretation of results. The software automatically evaluates the spine motions and extracts numerous features of interest. These can include flexibility, velocity, acceleration, symmetry, consistency, etc., in the axial, sagittal, and lateral planes of the body. Data can be viewed for an individual patient (Figure 4C), a population within a project, or across an organization for users with appropriate permissions. Data are stored in an access-controlled Open Web Application Security Project and National Institutes of Standards and Technology–compliant web portal, where various portions of collected and processed data can be evaluated with artificial intelligence or ML procedures to capture advanced interactions and provide input into biomechanical predictions of tissue-level forces.

The kinematic information derived from this motion capture system will also be used to inform a biomechanical model that predicts spine forces at the various levels of the lumbar spine. An ancillary study has been able to derive muscle activities from the trunk muscles, and this information is used in conjunction with spine imaging to predict patient-specific spine forces imposed on the discs.

## Future Directions and Conclusion

Biomechanical changes are intimately connected with LBP, regardless of the etiology of the pain. There is a concerted effort within BACPAC to characterize these changes at multiple scales of interest (whole-body motion, spinal kinematics, segmental kinematics, tissue-level biomechanical changes). The goals of this effort include developing mechanistic phenotypes for classifying and treating cLBP and tracking changes in functional biomechanics over time. Several novel technologies are being implemented in the consortium that have the potential to move biomechanical phenotyping of cLBP from the laboratory to the clinic and even to the home or workplace.

The comprehensive biomechanics data resource created through BACPAC will be shared broadly through a centralized data repository being developed by the Data Integration, Algorithm Development, and Operations Management Center. Consequently, the present work provides a helpful context for the spine research and clinical communities in interpreting and developing plans to use that resource as it becomes available. Additionally, we anticipate that the data and results from these studies will provide additional power in the context of the other deep phenotyping methods, such as spinal imaging, biospecimen analysis, and biobehavioral analysis, which are being advanced as part of the BACPAC effort.
